# Ramucirumab for advanced hepatocellular carcinoma in the current real world: a Japanese single-arm study post-REACH-2 (The R-evolution study)

**DOI:** 10.1007/s10637-024-01441-3

**Published:** 2024-06-06

**Authors:** Kazufumi Kobayashi, Sadahisa Ogasawara, Ei Itobayashi, Tomomi Okubo, Norio Itokawa, Kazuyoshi Nakamura, Michihisa Moriguchi, Shunji Watanabe, Masafumi Ikeda, Hidekatsu Kuroda, Tomokazu Kawaoka, Atsushi Hiraoka, Yutaka Yasui, Teiji Kuzuya, Rui Sato, Hiroaki Kanzaki, Keisuke Koroki, Masanori Inoue, Masato Nakamura, Soichiro Kiyono, Naoya Kanogawa, Takayuki Kondo, Shingo Nakamoto, Yoshihito Ozawa, Kaoru Tsuchiya, Masanori Atsukawa, Hiroshi Aikata, Takeshi Aramaki, Shiro Oka, Naoki Morimoto, Masayuki Kurosaki, Yoshito Itoh, Namiki Izumi, Naoya Kato

**Affiliations:** 1 Department of Gastroenterology, Graduate School of Medicine, Chiba University, Chiba, Japan; 2 Department of Gastroenterology, Asahi General Hospital, Asahi, Japan; 3 Division of Gastroenterology, Department of Internal Medicine, Nippon Medical School Chiba Hokusoh Hospital, Inzai, Japan; 4 Department of Gastroenterology, Chiba Cancer Center, Chiba, Japan; 5 Department of Gastroenterology and Hepatology, Kyoto Prefectural University of Medicine, Kyoto, Japan; 6 Division of Gastroenterology, Department of Medicine, Jichi Medical University, Shimotsuke, Japan; 7 Department of Hepatobiliary and Pancreatic Oncology, National Cancer Center Hospital East, Kashiwa, Japan; 8 Division of Gastroenterology and Hepatology, Department of Internal Medicine, Iwate Medical University, Yahaba, Japan; 9 Department of Gastroenterology and Metabolism, Hiroshima University Hospital, Hiroshima, Japan; 10 Gastroenterology Center, Ehime Prefectural Central Hospital, Matsuyama, Japan; 11 Department of Gastroenterology and Hepatology, Musashino Red Cross Hospital, Musashino, Japan; 12 Department of Gastroenterology and Hepatology, Fujita Health University, Toyoake, Japan; 13 Division of Interventional Radiology, Shizuoka Cancer Center, Nagaizumi, Japan; 14 Biostatistics Section, Clinical Research Center, Chiba University, Chiba, Japan; 15 Department of Gastroenterology, Hiroshima Prefectural Hospital, Hiroshima, Japan

**Keywords:** Hepatocellular carcinoma, Ramucirumab, REACH-2 trial, Real-world data

## Abstract

This study aimed to complement the results of the REACH-2 study by prospectively evaluating the safety and efficacy of ramucirumab in advanced hepatocellular carcinoma (HCC) in a real-world setting. This was an open-label, nonrandomized, multicenter, prospective study conducted at 13 institutions in Japan (jRCTs031190236). The study included Child–Pugh Class A patients with advanced HCC who had received pretreatment with atezolizumab plus bevacizumab (Atez/Bev) or lenvatinib. Ramucirumab was introduced as a second-line treatment after Atez/Bev or lenvatinib and as a third-line treatment after Atez/Bev and lenvatinib. Between May 2020 and July 2022, we enrolled 19 patients, including 17 who received ramucirumab. Additionally, seven patients received lenvatinib, another seven patients received Atez/Bev, and three patients received Atez/Bev followed by lenvatinib as prior treatment. The primary endpoint was a 6-month progression-free survival (PFS) rate, which was 14.3%. The median PFS and overall survival were 3.7 and 12.0 months, respectively. The most common grade ≥ 3 adverse events (AEs) were hypertension (23.5%), proteinuria (17.6%), and neutropenia (11.8%). The discontinuation rate due to AEs was 29.4%. Six patients progressed from Child–Pugh A to B after treatment with ramucirumab. Thirteen patients were eligible for post-ramucirumab treatment, including systemic therapy. Despite the limited number of patients, the efficacy of ramucirumab was comparable to that observed in the REACH-2 study when used after lenvatinib and Atez/Bev. However, the incidence of AEs was higher than that in the REACH-2 study.

**Supplementary information:**

The online version contains supplementary material available at 10.1007/s10637-024-01441-3.

## Introduction

Hepatocellular carcinoma (HCC), the seventh most common cancer and the third leading cause of cancer-related deaths, is a major global health concern [1]. Its primary origin is the liver, which is often associated with chronic liver diseases stemming from hepatitis B (HBV) and C virus (HCV) infections, alcohol abuse, or metabolic syndrome [2, 3]. HCC is prevalent in Asia, and cases have been increasing worldwide [1–4]. Despite global guidelines advocating early detection through surveillance of high-risk groups, many patients are still diagnosed at advanced stages [5–8]. Therefore, addressing the need for enhanced systemic therapies to improve outcomes in advanced HCC is critical.

Since the late 2010s, the treatment of advanced HCC has evolved significantly, starting with lenvatinib, which became the standard of care after the REFLECT trial showed its noninferiority to sorafenib. Further trials have highlighted the improved efficacy of lenvatinib [9]. The IMbrave 150 trial showed that the combination of atezolizumab and bevacizumab (Atez/Bev) outperformed sorafenib in terms of overall survival (OS), making it the preferred first-line treatment for advanced HCC [10]. The real-world efficacy of lenvatinib exceeds the results observed in the REFLECT trial [11, 12]. If immunotherapy is inadequate, lenvatinib is frequently used as a second-line treatment. In practical applications, lenvatinib is often preferred over sorafenib among tyrosine kinase inhibitors.

Ramucirumab, a vascular endothelial growth factor receptor (VEGFR)-2-targeting human immunoglobulin G monoclonal antibody, disrupts the VEGFR-2/vascular endothelial growth factor A (VEGF-A) interaction and reduces endothelial cell proliferation and migration [13–15]. Although the original REACH study did not show an OS benefit with best supportive care after progression on sorafenib, a subgroup analysis showed a significant benefit in patients with α-fetoprotein (AFP) levels of > 400 ng/mL [16]. This led to the REACH-2 trial, which confirmed the efficacy of ramucirumab in this specific patient population, leading to its approval as a standard second-line treatment for advanced HCC [17]. Because sorafenib is no longer the primary first-line therapy, the use of ramucirumab has adapted and is now typically administered after failure or intolerance to combination immunotherapy, such as Atez/Bev or lenvatinib. This evolving treatment context highlights the need to reevaluate the role of ramucirumab in advanced HCC. Therefore, this prospective study aimed to evaluate the safety and efficacy of ramucirumab in a contemporary real-world clinical setting for advanced HCC.

## Materials and methods

### Study design and patients

The R-evolution study, a multicenter, non-randomized, single-arm study, was conducted at 13 centers in Japan. Patients aged 20 years and older who were diagnosed with HCC confirmed by histology, cytology, or typical radiologic examination were enrolled. The study included patients who were ineligible for curative resection/local ablation and those ineligible for or unlikely to benefit from transcatheter arterial chemoembolization. Inclusion criteria encompassed a baseline Eastern Cooperative Oncology Group performance status of 0 or 1, Child–Pugh Class A, and measurable target lesions as defined by RECIST version 1.1, as well as a history of ineffective treatment or intolerance of first-line lenvatinib, first-line Atez/Bev, or Atez/Bev followed by lenvatinib. Eligibility also required adequate hematologic and organ function, as confirmed by baseline laboratory tests, including white blood cell count, neutrophil count, hemoglobin, platelet count, total bilirubin, aspartate aminotransferase, alanine aminotransferase, serum creatinine, and coagulation function. Major exclusion criteria were a history of other malignancy within the past 3 years, uncontrolled or significant cardiovascular disease, renal insufficiency requiring dialysis, active bacterial infection, grade ≥ 2 encephalopathy, a portal to main circulation shunt with hepatic encephalopathy, uncontrolled ascites, esophagogastric varices requiring treatment, history of variceal rupture, thromboembolism in the past 6 months, long-term antiplatelet therapy (except daily aspirin), and HBV DNA levels above the detection sensitivity without nucleotide analog treatment.

This study followed the International Conference on Harmonization Guidelines for Good Clinical Practice and the principles of the Declaration of Helsinki. All patients provided written informed consent. The institutional review board or ethics committee at each site approved the study protocol. The Certified Clinical Research Review Board at Chiba University approved the R-evolution trial on January 22, 2020, and approval was obtained from each participating institution (jRCTs031190236).

### Procedure

The patients received intravenous ramucirumab at 8 mg/kg every 2 weeks, similar to that in the REACH-2 study. Dose interruptions followed by dose reductions to 6 and 5 mg/kg were allowed for ramucirumab-related toxicities. Safety was assessed from the initiation of ramucirumab administration until study discontinuation. The investigators at each site assessed tumor response using the RECIST version 1.1 criteria. Treatment was continued until disease progression, unacceptable adverse events, patient withdrawal, or discontinuation was deemed in the best interest of the patient by the treating physician. Radiological assessment was performed 4 weeks after ramucirumab administration and then every 8 weeks during treatment. Eventually, treatment could be continued beyond progression if beneficial for the patient.

### Outcomes

The primary endpoint of the study was progression-free survival (PFS) at 6 months after ramucirumab initiation. The secondary efficacy endpoints were OS, PFS, and time to progression (TTP). The secondary safety endpoints were the incidence of adverse event (AE)s and discontinuation rate due to AEs. AEs were graded based on the National Cancer Institute Common Terminology Criteria for AEs version 4.0.

### Statistical analysis

The sample size was calculated as follows: assuming a 6-month expected PFS of 45% and a threshold PFS of 15%, a sample size of 27 participants would be required to provide 90% power, two-sided α = 5.0%, 12 months of enrollment, and 6 months of follow-up, calculated using the Fleming and Harrington method. We decided to enroll 30 participants to account for an expected dropout rate of 10%. The difference in the safety of ramucirumab between the three groups with each prior treatment will be evaluated as a secondary endpoint. At least one adverse event can be detected in six patients, which was expected to occur in > 30% of patients with a probability of > 75%. Therefore, we required at least six in each of the Atez/Bev, lenvatinib, and Atez/Bev and lenvatinib groups. The Kaplan–Meier was used to estimate the median OS, PFS, and TTP times and their 95% confidence intervals (CIs). All statistical analyses were performed using SAS statistical software, version 9.4 (SAS Institute, Cary, NC, RRID:SCR_008567).

## Results

### Patient characteristics

We obtained informed consent from 19 patients between March 2020 and June 2022. However, we excluded one patient because of deterioration of liver function prior to the start of treatment. In addition, another patient withdrew consent, thus only the remaining 17 patients received ramucirumab treatment (Supplementary Fig. 1). Table 1 shows the demographic and disease characteristics of the patients. The median age was 73 years, and 14 patients were male. At the initiation of ramucirumab, 6 and 11 patients had a Child–Pugh score of 5 and 6, respectively. Additionally, 14 patients were classified as Barcelona Clinic Liver Cancer stage C at treatment initiation. Median AFP level was 2,366 ng/mL (range, 551–50,270 ng/mL). Regarding prior treatment, 7 patients had received lenvatinib, 7 had received Atez/Bev, and 3 had received Atez/Bev followed by lenvatinib. Supplementary Table 1 shows the demographic and disease characteristics of each group, categorized by prior treatment. Supplementary Fig. 2 shows the changes of AFP levels of each group at the start of prior treatment and ramucirumab administration. All participants discontinued ramucirumab during the study. The median follow-up of the enrolled patients was 6.5 months (range, 2.8–14.4 months). Treatment was discontinued for various reasons during the observation period: 8 patients due to disease progression, 5 patients due to AEs leading to discontinuation, and 4 patients for other reasons, including decisions to withdraw consent.

**Table 1:** Patient characteristics

**Variable**	**Whole study population** **(*n*** **= 17)**
Age (years, median [range])	73 (42–89)
Sex, male (*n* [%])	14 (82.4%)
HBV positive (*n* [%])	8 (47.1%)
HCV positive (*n* [%])	4 (23.5%)
Alcohol Abuse (*n* [%])	5 (29.4%)
Child–Pugh score (*n* [%])	
5	6 (35.3%)
6	11 (64.7%)
ECOG-PS, 0 (*n* [%])	15 (88.2%)
Maximum intrahepatic tumor size ≥ 50 mm (*n* [%])	9 (52.9%)
Number of tumors, ≥ 8 (*n* [%])	4 (23.5%)
MVI (*n* [%])	8 (47.1%)
EHM (*n* [%])	9 (52.9%)
BCLC-C (*n* [%])	14 (82.4%)
AFP (ng/mL, median [range])	2,366 (551–50,270)
Prior treatment	
Lenvatinib (*n* [%])	7 (41.2%)
Atez/Bev (*n* [%])	7 (41.2%)
Atez/Bev followed by lenvatinib (*n* [%])	3 (17.6%)
Best radiological response of prior treatment	
Objective response	3 (17.6%)
Disease control	13 (76.5%)
Cause for the discontinuation of prior treatment, disease progression	13 (76.5%)

*HBV* hepatitis B virus, *HCV* hepatitis C virus, *ECOG-PS* Eastern Cooperative Oncology Group performance status, *MVI* macrovascular invasion, *EHM* extrahepatic metastasis, *BCLC* Barcelona Clinic Liver Cancer, *AFP* α-fetoprotein, *Atez/Bev* atezolizumab plus bevacizumab

### Efficacy

**Fig. 1:**
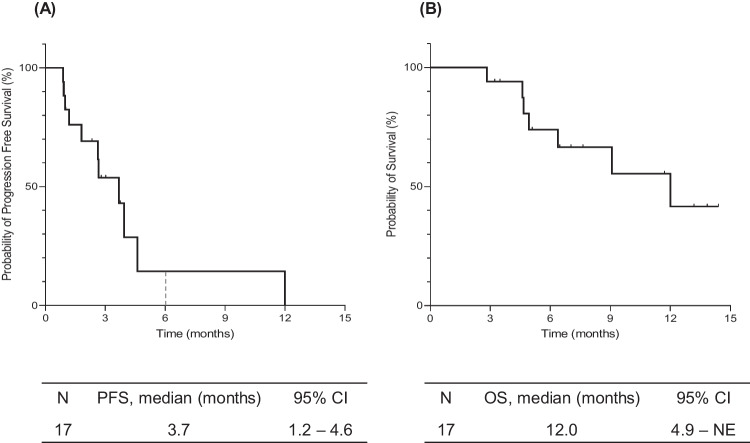
Survival analyses of patients with advanced hepatocellular carcinoma who received ramucirumab. **A** Progression-free survival. **B** Overall survival

**Fig. 2:**
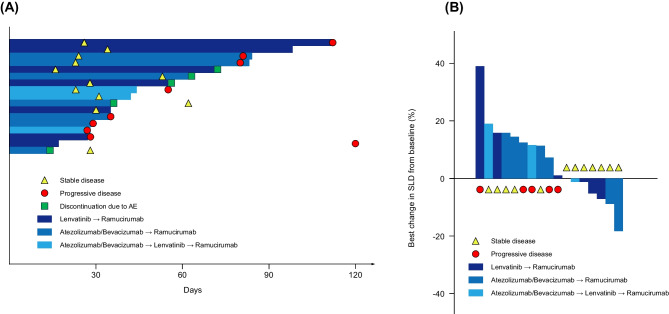
Efficacy of ramucirumab in 17 patients treated for advanced hepatocellular carcinoma. **A** Swimmer’s plot of treatment duration and the best response. **B** Best percent change in target lesions. Response was evaluated based on RECIST version 1.1. Abbreviation: SLD, sum of longest diameters

Figure 1 shows the Kaplan–Meier curves for PFS and OS. The 6-month PFS rate after ramucirumab initiation was 14.3% (95% CI, 0.9%–45.0%). The median PFS was 3.7 months (95% CI, 1.2–4.6) (Fig. 1A). The median OS was 12.0 months (95% CI, 4.9 months to not evaluable) (Fig. 1B). Figure 2A shows the swimmer plot visualizations showing the treatment duration and best responses for all patients. None of the study participants achieved a complete or partial response. Twelve patients had stable disease at their first radiological evaluation, whereas the remaining five patients had disease progression. The objective response and disease control rates were 0% and 70.6%, respectively. Notably, six patients remained on ramucirumab for > 2 months, whereas five patients discontinued treatment within the first month of administration. The treatment duration among the three groups classified based on their prior treatment regimens did not show significant differences. Figure 2B shows the best percentage change from baseline in target lesions for each patient. Although one patient had an approximately 40% increase in lesion size, most target lesion changes during treatment were generally within the 20% range. Supplementary Table 2 shows the detailed results of the prognostic analyses and best responses segmented by prior treatment. In the 12 patients where the best response to ramucirumab was disease control, 10 (83.3%) patients had disease control in prior treatments, while 2 (16.7%) patients experienced disease progression. On the other hand, among the 5 patients whose best response to ramucirumab was disease progression, 3 (60.0%) patients achieved disease control in prior treatments, while 2 (40.0%) patients showed disease progression (Supplementary Table 3).

### Safety

**Fig. 3:**
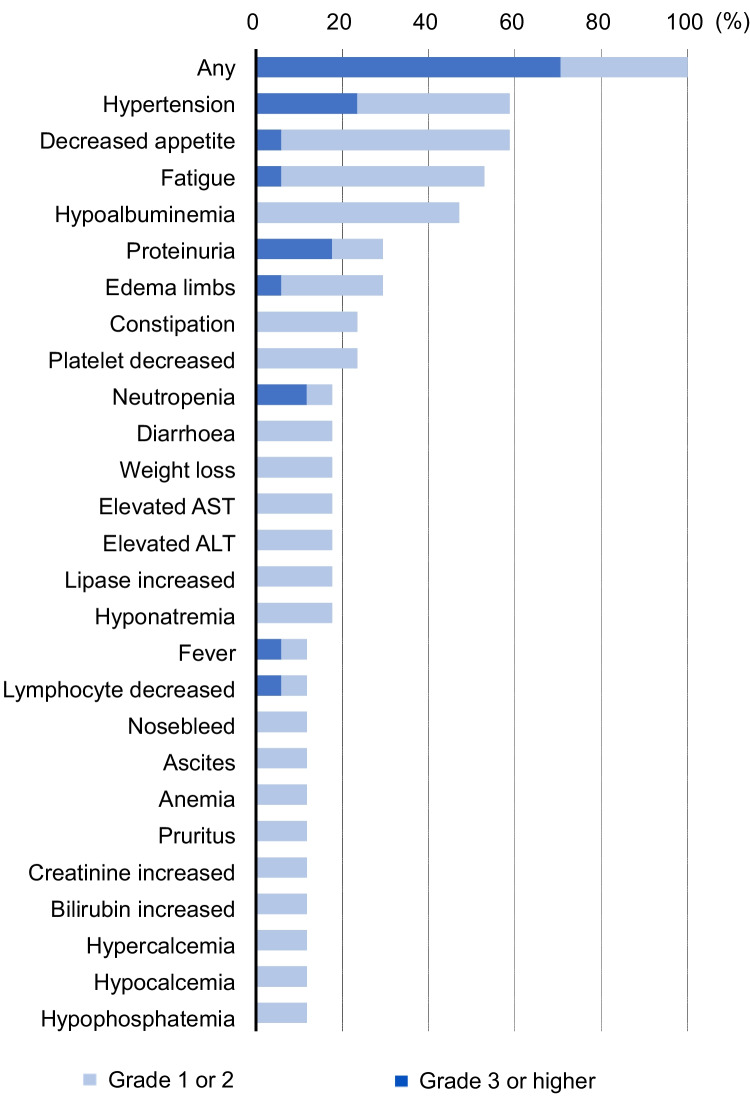
Incidence rates of adverse events in patients with advanced hepatocellular carcinoma receiving ramucirumab. Adverse events occurring ≥ 10% are presented in this figure

In this cohort, all patients experienced AEs of varying severity (Fig. 3 and Supplementary Table 4). Grade ≥ 3 AEs were observed in 12 patients (70.6%), with the most common being hypertension, proteinuria, and neutropenia in 23.5%, 17.6%, and 11.8% of patients. Regarding prior treatment category, the incidence of grade ≥ 3 AEs was highest in patients who received ramucirumab after lenvatinib at 85.7%, followed by 66.7% for patients treated with Atez/Bev followed by lenvatinib, and 57.1% for patients treated with Atez/Bev (Supplementary Table 5). Ramucirumab treatment had to be either interrupted or postponed in 10 patients (58.8%) during the study, dose reduction in 1 patient, postponement in 8 patients, and both dose reduction and postponement in 1 patient. The main causes for dose modification were decreased appetite, proteinuria, limb edema, and weight loss in two patients (11.8%). In addition, 5 patients (29.4%) had to discontinue treatment because of AEs, specifically proteinuria (2 patients), limb edema (1 patient), atrioventricular block (1 patient), and tumor lysis syndrome (1 patient).

### Transition of liver function and posttreatment after ramucirumab administration

**Fig. 4:**
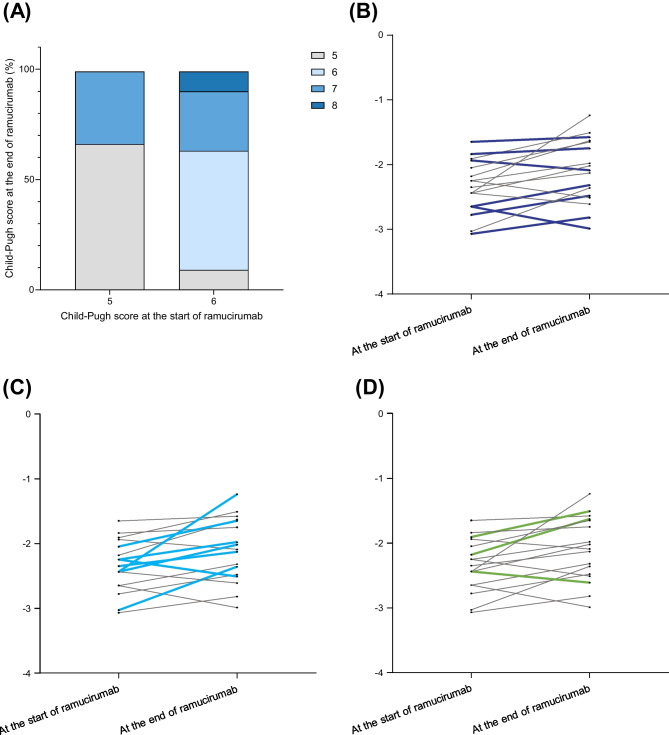
Transition of liver function reserve during ramucirumab treatment. **A** Correlation of Child–Pugh score at baseline and at end of ramucirumab treatment. **B** Transition of ALBI score. Purple line shows patients who received ramucirumab after lenvatinib. **C** Transition of ALBI score. Blue line shows patients who received ramucirumab after atezolizumab plus bevacizumab. **D** Transition of ALBI score. Green line shows patients who received ramucirumab after atezolizumab plus bevacizumab followed by lenvatinib. Abbreviation: ALBI, albumin bilirubin

Figure 4A shows the progression of Child–Pugh scores over the course of ramucirumab treatment. Among patients who started with a Child–Pugh score of 5, 66.7% (4 patients) remained in Child–Pugh class A, whereas 33.3% (2 patients) progressed to class B. Similarly, among patients with an initial score of 6, 63.6% (7 patients) remained in Child–Pugh class A, and 36.4% (4 patients) progressed to class B. The Child–Pugh scores of 10 patients did not change after ramucirumab treatment. Three patients had a one-point increase, three had a two-point increase, and one had a one-point decrease. Figure 4B–D shows the changes in albumin bilirubin (ALBI) levels at baseline and the end of ramucirumab treatment in each group classified based on the prior treatment, which indicates a lack of a consistent pattern of response. Supplementary Table 6 shows the options for subsequent anticancer treatments after ramucirumab administration. Most patients in the cohort (76.5%) could progress to subsequent treatments. Specifically, two patients progressed to hepatic arterial infusion chemotherapy, one patient received radiotherapy, and 10 patients received next-line systemic chemotherapy. The rates of progression to subsequent anticancer treatments were 85.7% for those who had previously received lenvatinib, 71.4% for those who had received Atez/Bev, and 66.7% for those who had received Atez/Bev and lenvatinib.

## Discussion

This prospective study was designed to evaluate the efficacy and safety of ramucirumab, particularly considering the shift in treatment of advanced HCC to the combination of Atez/Bev and lenvatinib. Although we were unable to reach its planned enrollment, it provided valuable data on the use of ramucirumab in the current treatment landscape for advanced HCC.

The primary endpoint of the study, the 6-month PFS rate for ramucirumab, was 14.3%, with a median PFS of 3.7 months, which was generally consistent with the efficacy of ramucirumab in the REACH-2 trial. The swimmer’s plot data showed that only 5 of 17 patients had disease progression at the first radiological evaluation, identifying them as “early progression patients,” indicating that ramucirumab achieved a degree of disease stabilization in a significant proportion of the participants. In actual clinical practice in Japan, ramucirumab will be administered often in the future as a follow-up treatment to either Atez/Bev or lenvatinib, mirroring the approach in this study. Although the combination of Atez/Bev is still awaiting approval, real-world data on ramucirumab in 79 patients who had received at least one or two prior therapies have been reported [18]. In this cohort, most patients had previously been treated with lenvatinib, showing a PFS of 3.2 months for those who received one or more prior treatments. Conversely, a retrospective study examining the effectiveness of ramucirumab after lenvatinib treatment in a small patient group reported a PFS of 2.0 months [19]. A recent retrospective study by Kuzuya et al., investigating the efficacy of ramucirumab following Atez/Bev, reported a TTP of 3.0 months [20]. Our prospective study’s findings align closely with these reports, indicating our results as reasonable and representative of the efficacy of ramucirumab in a real-world clinical setting.

The incidence of AEs during ramucirumab treatment in our study was significantly higher than that in the REACH-2 study. Specifically, we noted hypertension in 58.8% of all grades and 23.5% of grade ≥ 3 in our study compared with 25% of all grades and 13% of grade 3 or higher in REACH-2. In addition, proteinuria was common and a significant contributor to dose reduction or discontinuation of ramucirumab therapy. Although sorafenib, a tyrosine kinase inhibitor with VEGF inhibitory activity, has a relatively mild inhibition compared with other tyrosine kinase inhibitors approved for HCC [21–23], such as lenvatinib, our patient cohort had previously received bevacizumab, an anti-VEGF antibody, and lenvatinib as first- and second-line therapy. This prolonged and intense inhibition of the VEGF pathway may have increased the frequency or severity of AEs associated with VEGF inhibition. In our study, the high rate of adverse event-related discontinuation of ramucirumab and the treatment history described above may have influenced these results. In addition, the enrollment of only Japanese patients with advanced HCC who were older than those in the REACH-2 study may also be a contributing factor.

In this study, liver function slightly declined during ramucirumab treatment, which was consistent with previous findings [24]. Both Child–Pugh and ALBI scores remained largely unchanged from baseline to the end of therapy. Preservation of liver function for treating advanced HCC is equally important as tumor control in prolonging prognosis [25]. Currently, eight treatment regimens showed efficacy in international phase 3 trials for advanced HCC and are approved in Japan [26–28]. These regimens were specifically designed for patients with well-preserved liver function and are classified as Child–Pugh A. Thus, the preservation of liver function is a prerequisite for the approval of treatments for advanced HCC. Although several small studies have reported the safety and efficacy of existing treatments in Child–Pugh B patients, these results were not supported by a high level of evidence [11, 29–32]. To effectively sequence multiple approved agents, Child–Pugh A liver function should be preserved, thereby expanding the range of posttreatment options, whereas decline in liver function severely limits these options [25]. Notably, in our cohort, 13 of 17 patients could transition to some form of posttreatment.

Currently, combination immunotherapy, including Atez/Bev, is highly recommended as first-line treatment for advanced HCC [26–28]. For patients who are ineligible for this combination immunotherapy, existing VEGF tyrosine kinase inhibitors are generally considered, with preference for lenvatinib as a first-line alternative in real world practice in Japan [33]. However, the standard of care for patients who are either refractory or intolerant to combination immunotherapy remains unclear. In clinical practice, the median PFS with lenvatinib following Atez/Bev was between 2.0 and 6.1 months, indicating a trend toward shorter PFS compared with its use as first-line therapy [11, 34–37]. Furthermore, some studies, including our study, showed that the efficacy of other treatments, such as sorafenib, regorafenib, cabozantinib, and ramucirumab, after Atez/Bev does not exceed that of lenvatinib [20, 38, 39]. The exact role of ramucirumab in this setting remains to be determined. In addition, the use of ramucirumab in most countries and regions is limited to patients with AFP levels > 400 ng/mL, a criterion based on the REACH-2 study [17], which limits the patient population eligible for this treatment. The difficulty of enrollment in our study and the small size of most real-world clinical reports on this topic underscores this limitation. Unfortunately, our study results did not clarify the optimal positioning of ramucirumab in the latest treatment sequence for advanced HCC. Notably, ramucirumab showed efficacy comparable to that of REACH-2 in the most recent sequential treatment regimen, with preservation of liver function and a high rate of progression to subsequent treatments. As treatment options expand, a subset of patients with advanced HCC may be able to sequence multiple agents. In such scenarios, maintaining ramucirumab as an option for patients with AFP levels ≥ 400 ng/mL is critical to prolong patient survival.

Our study has several limitations that warrant mention. The primary constraint is the small sample size. Although the efficacy demonstrated in this study, which included a limited number of cases, is considered comparable to that of the REACH-2 trial, further validation of the efficacy of ramucirumab in patients with AFP ≥ 400 ng/mL in later-line treatment is necessary. This is particularly important within the current therapeutic framework, where Atez/Bev combination therapy is established as a first-line treatment. To confirm our findings, validation in a larger prospective cohort is essential. Furthermore, this study is limited to patients who have received prior treatment with Atez/Bev and lenvatinib. Patients who received first-line treatment with Atez/Bev followed by the first-generation tyrosine kinase inhibitor sorafenib and subsequently chose ramucirumab were not enrolled. This is due to the fact that in real-world clinical practice in Japan, lenvatinib is overwhelmingly the tyrosine kinase inhibitor of choice for first-line treatment [33]. Moreover, it is currently expected that the combination therapy of durvalumab and tremelimumab, durvalumab monotherapy, as well as regorafenib and cabozantinib, may be used prior to ramucirumab. Given the current diversification of sequential therapies for advanced HCC, prospectively verifying the safety of ramucirumab in all sequence patterns is challenging, which can be considered a limitation of this study. Lastly, the timing of imaging evaluations based on our trial design may have an impact on the efficacy assessment. In this study, the initial imaging evaluation was performed 4 weeks after the start of treatment. Compared to the REACH-2 trial, which conducted the initial evaluation 6 weeks after treatment initiation, there is a possibility of overestimating the efficacy. Our study prioritized the evaluation of the safety and efficacy of ramucirumab in real-world clinical practice in Japan, and therefore, we chose the commonly selected timepoint of 4 weeks after treatment initiation for the initial evaluation. However, this point should be taken into consideration when interpreting the results of this study.

In conclusion, our study supports the use of ramucirumab in advanced HCC, particularly after Atez/Bev or lenvatinib, highlighting its role in preserving liver function and providing additional treatment options for patients with advanced HCC. Further research and real-world data are essential to optimize advanced HCC treatment strategies.

### Supplementary information

Below is the link to the electronic supplementary material.

Supplementary file1 (PDF 399 kb)

## Data Availability

We included all generated or analyzed data in this study. Raw data were not publicly available to avoid compromising patient privacy or consent, but deidentified raw data were available upon reasonable request. Further inquiries may be directed to the corresponding author.

## References

[1] Sung H, Ferlay J, Siegel RL, Laversanne M, Soerjomataram I, Jemal A, Bray F (2021) Global cancer statistics 2020: GLOBOCAN estimates of incidence and mortality worldwide for 36 cancers in 185 countries. CA Cancer J Clin 71:209–249. 10.3322/caac.21660 33538338 10.3322/caac.21660

[2] Llovet JM, Kelley RK, Villanueva A et al (2021) Hepatocellular carcinoma. Nat Rev Dis Primers 7:6. 10.1038/s41572-020-00240-3 33479224 10.1038/s41572-020-00240-3 PMC13537433

[3] Vogel A, Meyer T, Sapisochin G, Salem R, Saborowski A (2022) Hepatocellular carcinoma. Lancet 400:1345–1362. 10.1016/S0140-6736(22)01200-4 36084663 10.1016/S0140-6736(22)01200-4

[4] Ogasawara S, Koroki K, Kanzaki H et al (2022) Changes in therapeutic options for hepatocellular carcinoma in Asia. Liver Int 42:2055–2066. 10.1111/liv.15101 34780081 10.1111/liv.15101

[5] European Association for the Study of the Liver. Electronic address: easloffice@easloffice.eu, European Association for the Study of the Liver (2018) EASL Clinical Practice Guidelines: management of hepatocellular carcinoma. J Hepatol 69:182–236. 10.1016/j.jhep.2018.03.019 29628281 10.1016/j.jhep.2018.03.019

[6] Heimbach JK, Kulik LM, Finn RS, Sirlin CB, Abecassis MM, Roberts LR, Zhu AX, Murad MH, Marrero JA (2018) AASLD guidelines for the treatment of hepatocellular carcinoma. Hepatology 67:358–380. 10.1002/hep.29086 28130846 10.1002/hep.29086

[7] Omata M, Cheng AL, Kokudo N et al (2017) Asia-Pacific clinical practice guidelines on the management of hepatocellular carcinoma: a 2017 update. Hepatol Int 11:317–370. 10.1007/s12072-017-9799-9 28620797 10.1007/s12072-017-9799-9 PMC5491694

[8] Kudo M, Kawamura Y, Hasegawa K et al (2021) Management of hepatocellular carcinoma in Japan: JSH consensus statements and recommendations 2021 update. Liver Cancer 10:181–223. 10.1159/000514174 34239808 10.1159/000514174 PMC8237791

[9] Kudo M, Finn RS, Qin S et al (2018) Lenvatinib versus sorafenib in first-line treatment of patients with unresectable hepatocellular carcinoma: a randomised phase 3 non-inferiority trial. Lancet 391:1163–1173. 10.1016/S0140-6736(18)30207-1 29433850 10.1016/S0140-6736(18)30207-1

[10] Finn RS, Qin S, Ikeda M et al (2020) Atezolizumab plus bevacizumab in unresectable hepatocellular carcinoma. N Engl J Med 382:1894–1905. 10.1056/NEJMoa1915745 32402160 10.1056/NEJMoa1915745

[11] Kobayashi K, Ogasawara S, Maruta S et al (2023) A prospective study exploring the safety and efficacy of lenvatinib for patients with advanced hepatocellular carcinoma and high tumor burden: the LAUNCH study. Clin Cancer Res 29:4760–4769. 10.1158/1078-0432.CCR-23-1462 37796614 10.1158/1078-0432.CCR-23-1462

[12] Llovet JM, Kudo M, Merle P et al (2023) Lenvatinib plus pembrolizumab versus lenvatinib plus placebo for advanced hepatocellular carcinoma (LEAP-002): a randomised, double-blind, phase 3 trial. Lancet Oncol 24:1399–1410. 10.1016/S1470-2045(23)00469-2 38039993 10.1016/S1470-2045(23)00469-2

[13] Spratlin JL, Cohen RB, Eadens M et al (2010) Phase I pharmacologic and biologic study of ramucirumab (IMC-1121B), a fully human immunoglobulin G1 monoclonal antibody targeting the vascular endothelial growth factor receptor-2. J Clin Oncol 28:780–787. 10.1200/JCO.2009.23.7537 20048182 10.1200/JCO.2009.23.7537 PMC2834394

[14] Zhu AX, Finn RS, Mulcahy M et al (2013) A phase II and biomarker study of ramucirumab, a human monoclonal antibody targeting the VEGF receptor-2, as first-line monotherapy in patients with advanced hepatocellular cancer. Clin Cancer Res 19:6614–6623. 10.1158/1078-0432.CCR-13-1442 24088738 10.1158/1078-0432.CCR-13-1442 PMC4795808

[15] Huang J, Zhang X, Tang Q, Zhang F, Li Y, Feng Z, Zhu J (2011) Prognostic significance and potential therapeutic target of VEGFR2 in hepatocellular carcinoma. J Clin Pathol 64:343–348. 10.1136/jcp.2010.085142 21270061 10.1136/jcp.2010.085142

[16] Zhu AX, Park JO, Ryoo BY et al (2015) Ramucirumab versus placebo as second-line treatment in patients with advanced hepatocellular carcinoma following first-line therapy with sorafenib (REACH): a randomised, double-blind, multicentre, phase 3 trial. Lancet Oncol 16:859–870. 10.1016/S1470-2045(15)00050-9 26095784 10.1016/S1470-2045(15)00050-9

[17] Zhu AX, Kang YK, Yen CJ et al (2019) Ramucirumab after sorafenib in patients with advanced hepatocellular carcinoma and increased α-fetoprotein concentrations (REACH-2): a randomised, double-blind, placebo-controlled, phase 3 trial. Lancet Oncol 20:282–296. 10.1016/S1470-2045(18)30937-9 30665869 10.1016/S1470-2045(18)30937-9

[18] Yasui Y, Kurosaki M, Tsuchiya K et al (2022) Real-world data on ramucirumab therapy including patients who experienced two or more systemic treatments: a multicenter study. Cancers 14:2975. 10.3390/cancers14122975 35740647 10.3390/cancers14122975 PMC9221496

[19] Hiraoka A, Kumada T, Tada T et al (2021) Therapeutic efficacy of ramucirumab after lenvatinib for post-progression treatment of unresectable hepatocellular carcinoma. Gastroenterol Rep (Oxf) 9:133–138. 10.1093/gastro/goaa042 34026220 10.1093/gastro/goaa042 PMC8128005

[20] Kuzuya T, Kawabe N, Hashimoto S et al (2022) Clinical outcomes of ramucirumab as post-treatment following atezolizumab/bevacizumab combination therapy in advanced hepatocellular carcinoma. Anticancer Res 42:1905–1910. 10.21873/anticanres.15667 35347009 10.21873/anticanres.15667

[21] Wilhelm SM, Carter C, Tang L et al (2004) BAY 43-9006 exhibits broad spectrum oral antitumor activity and targets the RAF/MEK/ERK pathway and receptor tyrosine kinases involved in tumor progression and angiogenesis. Cancer Res 64:7099–7109. 10.1158/0008-5472.CAN-04-1443 15466206 10.1158/0008-5472.CAN-04-1443

[22] Wilhelm SM, Dumas J, Adnane L, Lynch M, Carter CA, Schütz G, Thierauch KH, Zopf D (2011) Regorafenib (BAY 73-4506): a new oral multikinase inhibitor of angiogenic, stromal and oncogenic receptor tyrosine kinases with potent preclinical antitumor activity. Int J Cancer 129:245–255. 10.1002/ijc.25864 21170960 10.1002/ijc.25864

[23] Yamamoto Y, Matsui J, Matsushima T et al (2014) Lenvatinib, an angiogenesis inhibitor targeting VEGFR/FGFR, shows broad antitumor activity in human tumor xenograft models associated with microvessel density and pericyte coverage. Vasc Cell 6:18. 10.1186/2045-824X-6-18 25197551 10.1186/2045-824X-6-18 PMC4156793

[24] Kudo M, Galle PR, Brandi G et al (2021) Effect of ramucirumab on ALBI grade in patients with advanced HCC: results from REACH and REACH-2. JHEP Rep 3:100215. 10.1016/j.jhepr.2020.100215 33392490 10.1016/j.jhepr.2020.100215 PMC7772786

[25] Kobayashi K, Ogasawara S, Takahashi A et al (2022) Evolution of survival impact of molecular target agents in patients with advanced hepatocellular carcinoma. Liver Cancer 11:48–60. 10.1159/000519868 35222507 10.1159/000519868 PMC8820147

[26] Bruix J, Chan SL, Galle PR, Rimassa L, Sangro B (2021) Systemic treatment of hepatocellular carcinoma: an EASL position paper. J Hepatol 75:960–974. 10.1016/j.jhep.2021.07.004 34256065 10.1016/j.jhep.2021.07.004

[27] Singal AG, Llovet JM, Yarchoan M et al (2023) AASLD practice guidance on prevention, diagnosis, and treatment of hepatocellular carcinoma. Hepatology 78:1922–1965. 10.1097/HEP.0000000000000466 37199193 10.1097/HEP.0000000000000466 PMC10663390

[28] Hasegawa K, Takemura N, Yamashita T et al (2023) Clinical practice guidelines for hepatocellular carcinoma: the Japan Society of Hepatology 2021 version (5th JSH-HCC guidelines). Hepatol Res 53:383–390 36826411 10.1111/hepr.13892

[29] Ogasawara S, Chiba T, Ooka Y et al (2015) Sorafenib treatment in Child-Pugh A and B patients with advanced hepatocellular carcinoma: safety, efficacy and prognostic factors. Invest New Drugs 33:729–739. 10.1007/s10637-015-0237-3 25861764 10.1007/s10637-015-0237-3

[30] Maruta S, Ogasawara S, Ooka Y et al (2020) Potential of lenvatinib for an expanded indication from the REFLECT trial in patients with advanced hepatocellular carcinoma. Liver Cancer 9:382–396. 10.1159/000507022 32999866 10.1159/000507022 PMC7506220

[31] Kudo M, Matilla A, Santoro A et al (2021) CheckMate 040 cohort 5: a phase I/II study of nivolumab in patients with advanced hepatocellular carcinoma and Child-Pugh B cirrhosis. J Hepatol 75:600–609. 10.1016/j.jhep.2021.04.047 34051329 10.1016/j.jhep.2021.04.047

[32] D’Alessio A, Fulgenzi CAM, Nishida N et al (2022) Preliminary evidence of safety and tolerability of atezolizumab plus bevacizumab in patients with hepatocellular carcinoma and Child-Pugh A and B cirrhosis: a real-world study. Hepatology 76:1000–1012. 10.1002/hep.32468 35313048 10.1002/hep.32468 PMC9790703

[33] Asaoka Y, Tateishi R, Yamada Y et al (2023) Real world data of systemic therapy for hepatocellular carcinoma in Japan: HERITAGE study. J Clin Oncol 41:510. 10.1200/jco.2023.41.4_suppl.510 10.1200/jco.2023.41.4_suppl.510

[34] Hiraoka A, Kumada T, Tada T et al (2023) Lenvatinib as second-line treatment after atezolizumab plus bevacizumab for unresectable hepatocellular carcinoma: clinical results show importance of hepatic reserve function. Oncology 101:624–633. 10.1159/000531316 37307798 10.1159/000531316

[35] Yano S, Kawaoka T, Yamasaki S et al (2023) Therapeutic efficacy and safety of lenvatinib after atezolizumab plus bevacizumab for unresectable hepatocellular carcinoma. Cancers (Basel) 15:5406. 10.3390/cancers15225406 38001666 10.3390/cancers15225406 PMC10670624

[36] Yoo C, Kim JH, Ryu MH et al (2021) Clinical outcomes with multikinase inhibitors after progression on first-line atezolizumab plus bevacizumab in patients with advanced hepatocellular carcinoma: a multinational multicenter retrospective study. Liver Cancer 10:107–114. 10.1159/000512781 33977087 10.1159/000512781 PMC8077483

[37] Chen CT, Feng YH, Yen CJ, Chen SC, Lin YT, Lu LC, Hsu CH, Cheng AL, Shao YY (2022) Prognosis and treatment pattern of advanced hepatocellular carcinoma after failure of first-line atezolizumab and bevacizumab treatment. Hepatol Int 16:1199–1207. 10.1007/s12072-022-10392-x 35986846 10.1007/s12072-022-10392-x

[38] Falette-Puisieux M, Nault JC, Bouattour M et al (2023) Beyond atezolizumab plus bevacizumab in patients with advanced hepatocellular carcinoma: overall efficacy and safety of tyrosine kinase inhibitors in a real-world setting. Ther Adv Med Oncol 15:17588359231189424. 10.1177/17588359231189425 37547443 10.1177/17588359231189425 PMC10399252

[39] Shimose S, Sugimoto R, Hiraoka A et al (2023) Significance of ramucirumab following atezolizumab plus bevacizumab therapy for hepatocellular carcinoma using real-world data. Hepatol Res 53:116–126. 10.1111/hepr.13852 36316794 10.1111/hepr.13852

