# Supplementary material for: Ramucirumab for advanced hepatocellular carcinoma in the current real world: a Japanese single-arm study post-REACH-2 (The R-evolution study)
Source: Invest New Drugs. 2024 Jun 6;42(4):394–404. doi: 10.1007/s10637-024-01441-3 (PMC11327193; doi:10.1007/s10637-024-01441-3)
Supplement: Supplementary file 1 — Supplementary file1 (PDF 399 kb) [file 10637_2024_1441_MOESM1_ESM.pdf]

Supplementary Materials

This appendix has been provided by the authors to give reader additional information about their work.

Contents
Supplementary Fig. 1. Patient flow of the R-evolution study
Supplementary Table 1. Patient characteristics for each prior treatment group
Supplementary Fig. 2. Changes of AFP levels at the start of the prior treatment and ramucirumab administration
Supplementary Table 2. Clinical outcomes and radiological assessments
Supplementary Table 3. Correlation between the clinical course of prior treatment and the effect of ramucirumab
Supplementary Table ~~3~~4. Adverse events during the study period
Supplementary Table 45. Adverse events for each prior treatment group
Supplementary Table ~~5~~6. Subsequent anti-cancer treatment after ramucirumab

Supplementary Fig. 1. Patient flow of the R-evolution study

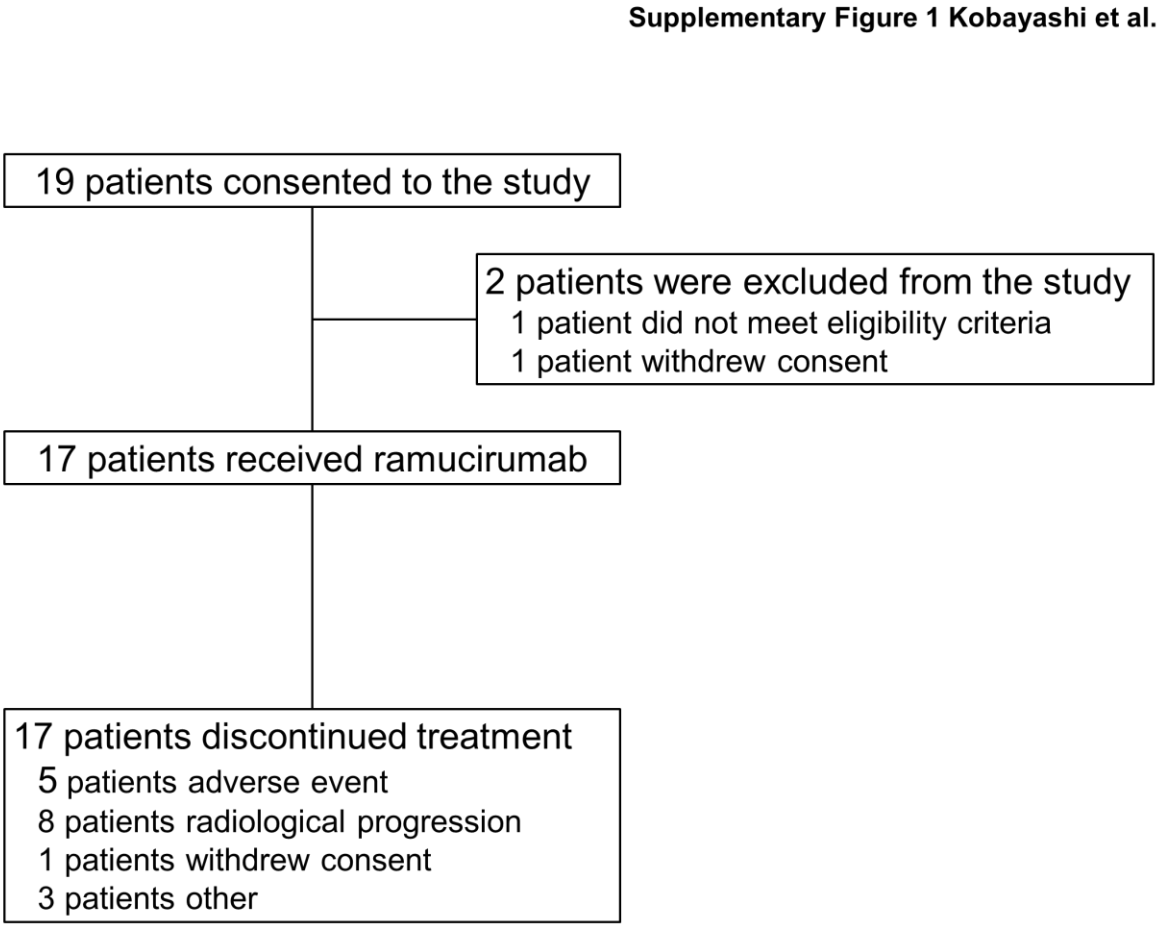

Supplementary Table 1. Patient characteristics for each prior treatment group

Variable	Prior treatment		
	**Lenvatinib (n = 7)**	**Atez/Bev (n = 7)**	**Atez/Bev → Lenvatinib (n = 3)**
Age (years, median [range])	73 (47–79)	75 (58–89)	71 (42–79)
Sex, male (n [%])	6 (85.7%)	5 (71.4%)	3 (100.0%)
HBV positive (n [%])	3 (42.9%)	3 (42.9%)	2 (66.7%)
HCV positive (n [%])	0	4 (57.1%)	0
Alcohol Abuse (n [%])	2 (28.6%)	2 (28.6%)	1 (33.3%)
Child-Pugh score (n [%])			
5	3 (42.9%)	2 (28.6%)	1 (33.3%)
6	4 (57.1%)	5 (71.4%)	2 (66.7%)
ECOG-PS, 0 (n [%])	6 (85.7%)	6 (85.7%)	3 (100.0%)
Maximum intrahepatic tumor size ≥50 mm (n [%])	5 (71.4%)	2 (28.6%)	2 (66.7%)
Number of tumors, ≥8 (n [%])	2 (28.6%)	0	2 (66.7%)
MVI (n [%])	2 (28.6%)	4 (57.1%)	2 (66.7%)
EHM (n [%])	4 (57.1%)	4 (57.1%)	1 (33.3%)
BCLC-C	6 (85.7%)	6 (85.7%)	2 (66.7%)
AFP (ng/mL, median [range])	2,820 (628–50,270)	1,643 (551–15,563)	16,406 (2,366–28,370)
Best radiological response of prior treatment			
Objective response	1 (14.3%)	1 (14.3%)	1 (33.3%)
Disease control	5 (71.4%)	5 (71.4%)	3 (100.0%)
Cause for the discontinuation of prior treatment, disease progression	4 (57.1%)	7 (100.0%)	2 (66.7%)

Abbreviation: HBV, hepatitis B virus; HCV, hepatitis C virus; ECOG-PS, Eastern Cooperative Oncology Group performance status; MVI, macrovascular invasion; EHM, extrahepatic metastasis; BCLC, Barcelona Clinic liver cancer; AFP, α-fetoprotein; Atez/Bev, atezolizumab plus bevacizumab

Supplementary Fig. 2. Changes of AFP levels at the start of the prior treatment and ramucirumab administration

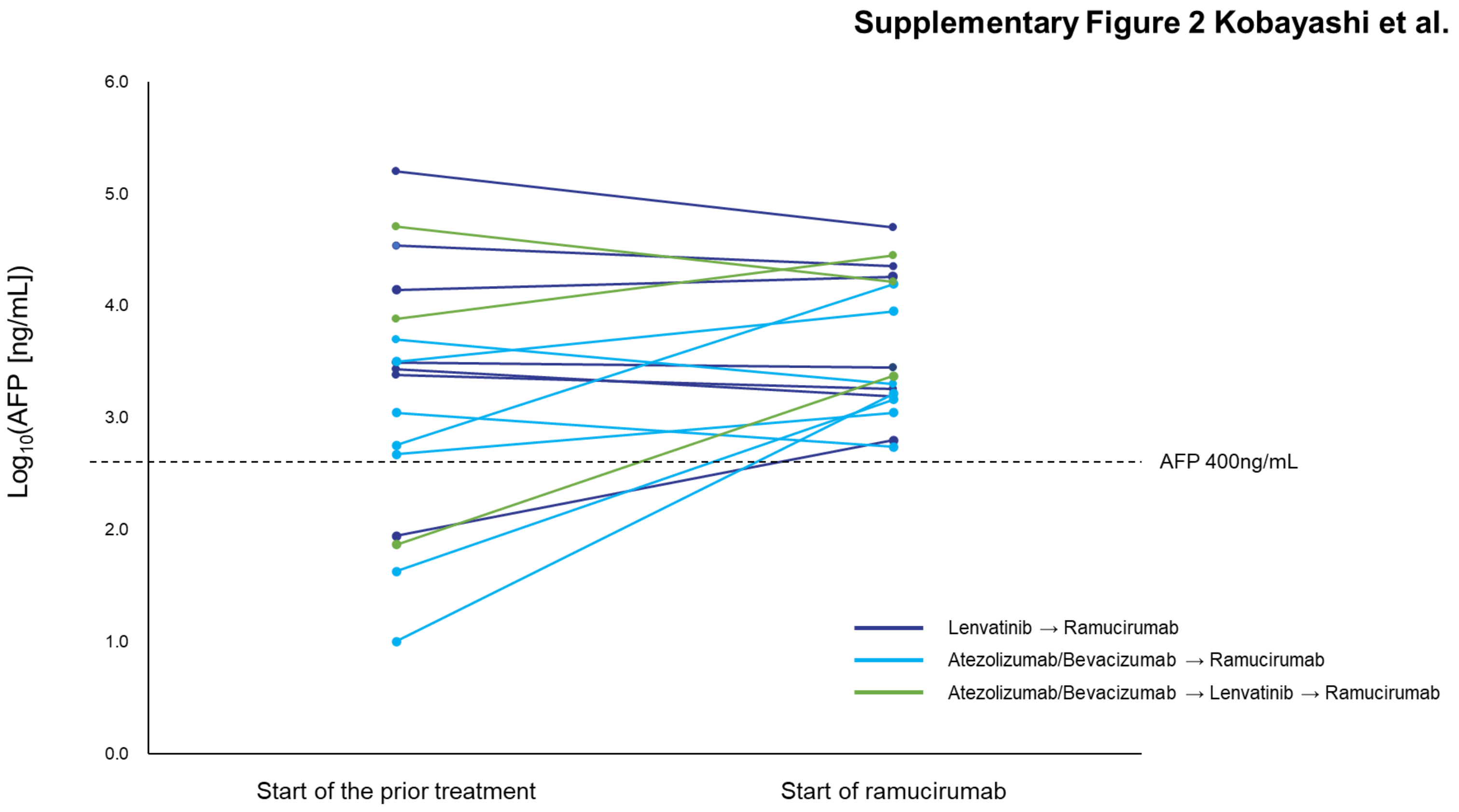

Abbreviation: AFP, α-fetoprotein;

Supplementary Table 2. Clinical outcomes and radiological assessments

Variable	Whole study population (n = 17)	Prior treatment		
		Lenvatinib (n = 7)	Atez/Bev (n = 7)	Atez/Bev → Lenvatinib (n = 3)
OS (months [95% CI])	12.0 (4.9 – NE)	Not reached	Not reached	4.9 (4.4 – 5.4)
PFS, (months [95% CI])	3.7 (1.2 – 4.6)	3.9 (3.6 – 4.2)	2.7 (1.1 – 4.3)	1.8 (0.4 – 3.2)
Best response, (n [%])				
CR	0	0	0	0
PR	0	0	0	0
SD	12 (70.6%)	5 (71.4%)	5 (71.4%)	2 (66.7%)
PD	5 (29.4%)	2 (28.6%)	2 (28.6%)	1 (33.3%)
Objective response	0	0	0	0
Disease control	12 (70.6%)	5 (71.4%)	5 (71.4%)	2 (66.7%)

Abbreviation: OS, overall survival; PFS, progression free survival; CR, complete response; PR, partial response; SD, stable disease; PD, progression disease; CI, confidence interval; Atez/Bev, atezolizumab plus bevacizumab; NE, not evaluable

Supplementary Table 3. Correlation between the clinical course of prior treatment and the effect of ramucirumab

	**Best radiological response of ramucirumab**	
	**Disease control**	**PD**
Best radiological response of prior treatment		
Objective response	1 (8.3%)	2 (40.0%)
Disease control	10 (83.3%)	3 (60.0%)
PD	2 (16.7%)	2 (40.0%)
Cause for the discontinuation of prior treatment, disease progression	9 (75.0%)	4 (80.0%)

Abbreviation: PD, progression disease

Supplementary Table 34. Adverse events during the study period

Adverse events	Occurrence of AE		Dose modification due to AE	Treatment discontinuation due to AE
	Any	≥Grade 3		
Any	17 (100.0%)	12 (70.6%)	15 (88.2%)	5 (29.4%)
Hypertension	10 (58.8%)	4 (23.5%)	1 (5.9%)	0
Decreased appetite	10 (58.8%)	1 (5.9%)	2 (11.8%)	0
Fatigue	9 (52.9%)	1 (5.9%)	1 (5.9%)	0
Hypoalbuminemia	8 (47.1%)	0	0	0
Proteinuria	5 (29.4%)	3 (17.6%)	2 (11.8%)	2 (11.8%)
Edema limbs	5 (29.4%)	1 (5.9%)	2 (11.8%)	1 (5.9%)
Constipation	4 (23.5%)	0	0	0
Platelet decreased	4 (23.5%)	0	0	0
Neutropenia	3 (17.6%)	2 (11.8%)	1 (5.9%)	0
Diarrhoea	3 (17.6%)	0	1 (5.9%)	0
Weight loss	3 (17.6%)	0	2 (11.8%)	0
Elevated AST	3 (17.6%)	0	0	0
Elevated ALT	3 (17.6%)	0	0	0
Lipase increased	3 (17.6%)	0	0	0
Hyponatremia	3 (17.6%)	0	0	0
Fever	2 (11.8%)	1 (5.9%)	1 (5.9%)	0
Lymphocyte decreased	2 (11.8%)	1 (5.9%)	0	0
Nosebleed	2 (11.8%)	0	0	0
Ascites	2 (11.8%)	0	1 (5.9%)	0
Anemia	2 (11.8%)	0	0	0
Pruritus	2 (11.8%)	0	0	0
Creatinine increased	2 (11.8%)	0	0	0
Bilirubin increased	2 (11.8%)	0	0	0
Hypercalcemia	2 (11.8%)	0	0	0
Hypocalcemia	2 (11.8%)	0	0	0
Hypophosphatemia	2 (11.8%)	0	0	0
Atrioventricular block	1 (5.9%)	1 (5.9%)	0	1 (5.9%)
Tumor lysis syndrome	1 (5.9%)	1 (5.9%)	1 (5.9%)	1 (5.9%)

Abbreviation: AST, aspartate aminotransferase; ALT, alanine aminotransferase; AE, adverse event

Supplementary Table 45. Adverse events for each prior treatment group

Adverse events	Prior treatment					
	Lenvatinib (n = 7)		Atez/Bev (n = 7)		Atez/Bev → Lenvatinib (n = 3)	
	Any	≥Grade 3	Any	≥Grade 3	Any	≥Grade 3
Any	7 (100.0%)	6 (85.7%)	7 (100.0%)	4 (57.1%)	3 (100.0%)	2 (66.7%)
Hypertension	4 (57.1%)	1 (14.3%)	5 (71.4%)	2 (28.6%)	1 (33.3%)	1 (33.3%)
Decreased appetite	5 (71.4%)	1 (14.3%)	4 (57.1%)	0	1 (33.3%)	0
Fatigue	5 (71.4%)	1 (14.3%)	2 (28.6%)	0	2 (66.7%)	0
Hypoalbuminemia	3 (42.9%)	0	3 (42.9%)	0	2 (66.7%)	0
Proteinuria	0	0	3 (42.9%)	2 (28.6%)	2 (66.7%)	1 (33.3%)
Edema limbs	2 (28.6%)	0	3 (42.9%)	1 (14.3%)	0	0
Constipation	2 (28.6%)	0	2 (28.6%)	0	0	0
Platelet decreased	2 (28.6%)	0	2 (28.6%)	0	0	0
Neutropenia	2 (28.6%)	1 (14.3%)	0	0	1 (33.3%)	1 (33.3%)
Diarrhoea	2 (28.6%)	0	0	0	1 (33.3%)	0
Weight loss	3 (42.9%)	0	0	0	0	0
Elevated AST	2 (28.6%)	0	0	0	1 (33.3%)	0
Elevated ALT	2 (28.6%)	0	0	0	1 (33.3%)	0
Lipase increased	2 (28.6%)	0	1 (14.3%)	0	0	0
Hyponatremia	2 (28.6%)	0	1 (14.3%)	0	0	0
Fever	1 (14.3%)	0	0	0	1 (33.3%)	1 (33.3%)
Lymphocyte decreased	0	0	0	0	2 (66.7%)	1 (33.3%)
Nosebleed	1 (14.3%)	0	1 (14.3%)	0	0	0
Ascites	0	0	2 (28.6%)	0	0	0
Anemia	0	0	1 (14.3%)	0	1 (33.3%)	0
Pruritus	0	0	1 (14.3%)	0	1 (33.3%)	0
Creatinine increased	0	0	1 (14.3%)	0	1 (33.3%)	0
Bilirubin increased	1 (14.3%)	0	1 (14.3%)	0	0	0
Hypercalcemia	1 (14.3%)	0	0	0	1 (33.3%)	0
Hypocalcemia	1 (14.3%)	0	1 (14.3%)	0	0	0
Hypophosphatemia	2 (28.6%)	0	0	0	0	0
Atrioventricular block	1 (14.3%)	1 (14.3%)	0	0	0	0
Tumor lysis syndrome	1 (14.3%)	1 (14.3%)	0	0	0	0

Abbreviation: AST, aspartate aminotransferase; ALT, alanine aminotransferase; Atez/Bev, atezolizumab plus bevacizumab

Supplementary Table ~~5~~6. Subsequent anti-cancer treatment after ramucirumab

Variable	Whole study population (n = 17)	Prior treatment		
		**Lenvatinib (n = 7)**	**Atez/Bev (n = 7)**	**Atez/Bev → Lenvatinib (n = 3)**
Any treatment	13 (76.5%)	6 (85.7%)	5 (71.4%)	2 (66.7%)
Any IO	2 (11.8%)	2 (28.6%)	0	0
Atez/Bev	2 (11.8%)	2 (28.6%)	0	0
Others	0	0	0	0
TKI/VEGF	8 (47.1%)	3 (42.9%)	4 (57.1%)	1 (33.3%)
Sorafenib	5 (29.4%)	3 (42.9%)	2 (28.6%)	0
Regorafenib	1 (5.9%)	0	1 (14.3%)	0
Lenvatinib	2 (11.8%)	0	1 (14.3%)	1 (33.3%)
Cabozantinib	0	0	0	0
TACE	0	0	0	0
HAIC	2 (11.8%)	1 (14.3%)	0	1 (33.3%)
Surgical resection	0	0	0	0
Radiation	1 (5.9%)	0	1 (14.3%)	0

Abbreviation: IO, immuno-oncology; Atez/Bev, atezolizumab plus bevacizumab; TKI, tyrosine kinase inhibitor; VEGF, vascular endothelial growth factor; TACE, transcatheter arterial chemoembolization; HAIC, hepatic arterial infusion chemotherapy
